# Non-coding RNAs and ferroptosis: potential implications for cancer therapy

**DOI:** 10.1038/s41418-022-00998-x

**Published:** 2022-04-14

**Authors:** Amar Balihodzic, Felix Prinz, Michael A. Dengler, George A. Calin, Philipp J. Jost, Martin Pichler

**Affiliations:** 1 Department of Internal Medicine, Division of Oncology, Medical University of Graz, 8036 Graz, Austria; 2 Research Unit “Non-Coding RNAs and Genome Editing in Cancer”, Division of Oncology, Medical University of Graz, 8036 Graz, Austria; 3 Department of Translational Molecular Pathology, The University of Texas MD Anderson Cancer Center, Houston, TX 77030 USA; 4 Medical Department III for Hematology and Oncology, TUM School of Medicine, Technical University of Munich, Munich, Germany; 5 Department of Experimental Therapeutics, The University of Texas MD Anderson Cancer Center, Houston, TX 77030 USA

**Keywords:** Tumour biomarkers, Oncogenes

## Abstract

Ferroptosis is a recently defined form of regulated cell death, which is biochemically and morphologically distinct from traditional forms of programmed cell death such as apoptosis or necrosis. It is driven by iron, reactive oxygen species, and phospholipids that are oxidatively damaged, ultimately resulting in mitochondrial damage and breakdown of membrane integrity. Numerous cellular signaling pathways and molecules are involved in the regulation of ferroptosis, including enzymes that control the cellular redox status. Alterations in the ferroptosis-regulating network can contribute to the development of various diseases, including cancer. Evidence suggests that ferroptosis is commonly suppressed in cancer cells, allowing them to survive and progress. However, cancer cells which are resistant to common chemotherapeutic drugs seem to be highly susceptible to ferroptosis inducers, highlighting the great potential of pharmacologic modulation of ferroptosis for cancer treatment. Non-coding RNAs (ncRNAs) are considered master regulators of various cellular processes, particularly in cancer where they have been implicated in all hallmarks of cancer. Recent work also demonstrated their involvement in the molecular control of ferroptosis. Hence, ncRNA-based therapeutics represent an exciting alternative to modulate ferroptosis for cancer therapy. This review summarizes the ncRNAs implicated in the regulation of ferroptosis in cancer and highlights their underlying molecular mechanisms in the light of potential therapeutic applications.

## Facts

- Ferroptosis is a unique, iron-dependent, oxidative form of regulated cell death.
- Ferroptosis is frequently suppressed in cancer supporting its growth and progression.
- Ferroptotic rich regulatory network is essentially modulated by ncRNAs.
- NcRNA-based therapeutics targeting ferroptosis is a promising novel anti-cancer therapy.

## Open questions

- What is the relationship between ncRNAs, ferroptosis and other forms of regulated cell death in cancer?
- Are individual ncRNAs potential molecular markers of ferroptosis that could be used in living cells and tissues?
- What are the optimal delivery systems of novel ncRNAs-therapeutics, particularly for efficient intracellular uptake and controlled release?
- Would potential combination of available drugs with novel ncRNA-therapeutics modulating ferroptosis result in an improved cancer treatment?

## Introduction

### Non-coding RNAs: master regulators of cellular processes

Non-coding RNAs (NcRNAs) are a miscellaneous group of non-coding transcripts with limited protein-coding potential that perform important cellular functions through different molecular mechanisms [1]. Initially, it was thought that they are functionally irrelevant. However, as a myriad of functional ncRNAs were identified and characterized, the central dogma of proteins being the functional end product of gene expression has drastically changed [2].

Broadly spoken, they can either be subdivided into short and long ncRNAs (a general cut off value is 200 nucleotides in length), or subdivided due to their biological roles [3]. Three major classes of functional ncRNAs are short microRNAs (miRNAs), long ncRNAs (lncRNAs), and circular RNAs (circRNAs). Depending on their intrinsic features, they may show tissue- and/or disease-specificity and may be detected in all body fluids making them interesting for their potential utilization as biomarkers [4]. In addition, ncRNAs are frequently deregulated in various diseases, including cancer [5]. In many instances their involvement in drug resistance in cancers has been reported [6–9]. Thus, targeting of ncRNAs might be a promising therapeutic option to modulate drug resistance-promoting pathways in cancer cells and improve the outcome of patients [10].

### Ferroptosis: the molecular mechanisms of a recently identified form of cell death

Ferroptosis is an iron-dependent, oxidative form of cell death that is biochemically and morphologically different from other types of regulated cell death [11–13]. Ferroptosis is caused by excessive oxidative destruction (peroxidation) of lipids in the cellular membranes. The process relies on iron, reactive oxygen species (ROS), and phospholipids containing polyunsaturated fatty acids (PUFAs) [14–16].

Lipid peroxidation occurs when a bisallylic hydrogen atom, located between two carbon–carbon double bonds, is removed from the PUFAs in the membrane phospholipids. The result is formation of a carbon-centered phospholipid radical, phospholipid peroxyl radical, and phospholipid peroxides, a form of lipid ROS. Phospholipid peroxides can react with iron to generate free alkoxyl and peroxyl radicals [17]. The requirement of iron in this form of cell death inspired the term ferroptosis [12]. If not converted to its corresponding alcohol, phospholipid peroxides, together with phospholipid free radicals, promote further phospholipid peroxide formation via the processes of hydrogen removal and reaction with oxygen. It is the unrestrained lipid peroxidation that is considered to be the hallmark of ferroptosis [18].

Some first hints of ferroptosis-like death have been observed in the middle of 20-th century in studies investigating metabolism and neuronal cell death. The earliest reports attributed ferroptosis either to other forms of regulated cell death, or it was not recognized as being biologically significant. It was not interpreted as sufficient evidence for a distinct cell death until early 2000s when the Stockwell lab conducted screening of lethal compounds in RAS-transformed cancer cells. They identified erastin and RAS synthetic lethal 3 (RSL3) as inducers of non-apoptotic, iron-dependent cell death preventable by iron chelators and lipophilic antioxidants [19, 20]. The following findings, including the mechanism of action of erastin and RSL3, lead to the idea of a unique regulated cell death form. The term ferroptosis was introduced in 2012, and, thus, the field of ferroptosis research is rather officially young [12]. Despite being frequently cited as a new type of cell death, ferroptosis may actually be considered the oldest and evolutionary most conserved form of regulated cell death owing to its simple molecular requirements of iron and oxygen. In fact, ferroptosis-like death has been observed in less-complex species including protozoa, prokaryotes, fungi and plants [21–24].

### Ferroptosis initiation and regulation

**Fig. 1:**
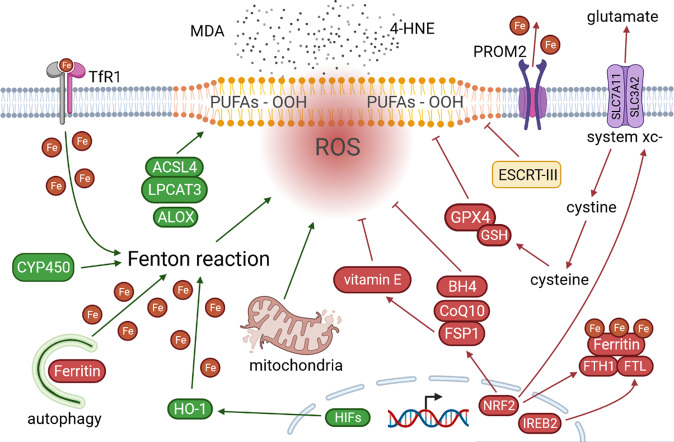
Ferroptosis mechanisms. Ferroptosis can be initiated by non-enzymatic and enzymatic processes. The non-enzymatic process includes Fenton reaction, where iron and hydrogen peroxide react and form free radicals. Various enzymes and proteins drive ferroptosis via increasing iron availability and enhancing free radical formation (CYP450, HO-1, HIFs), and performing important roles in biosynthesis and oxidation of PUFAs (ACSL4, LPCAT3, ALOX). In contrast, ferroptosis is suppressed by several enzymes and cofactors with antioxidant functions (GPX4, GSH, FSP1, CoQ10, BH4) and system x_c_^-^ cystine/glutamate antiporter (SLC7A11 and SLC3A2) that import cystine which is necessary for the biosynthesis of antioxidant enzymes. Consequences of ferroptosis are various harmful breakdown products (e.g., MDA and 4-HNE), modified and oxidized proteins (e.g., reparatory ESCRT-III), damaged mitochondria and eventual disruption of membrane integrity. MDA, malondialdehyde; 4-HNE, 4-hydroxy-2-nonenal; TfR1, transferrin receptor 1; PUFAs-OOH, polyunsaturated fatty acid peroxides; ROS, reactive oxygen species; PROM2, prominin 2; system xc-, cystine/glutamate antiporter; SLC7A11, solute carrier family 7 member 11; SLC3A2, solute carrier family 3 member 2; ESCRT-III, endosomal sorting complexes required for transport-III; Fe, ferrum (iron); ACSL4, acyl-CoA synthetase long-chain family member 4; LPCAT3, lysophosphatidylcholine acyltransferase 3; ALOX, lipoxygenase; CYP450, cytochrome P450; GPX4, glutathione peroxidase 4; GSH, glutathione; BH4, tetrahydrobiopterin; CoQ10, coenzyme Q10; FSP1, ferroptosis suppressor protein 1; FTH1, ferritin heavy chain 1; FTL, ferritin light chain; NRF2, nuclear factor E2-related factor 2; IREB2, iron-responsive element binding protein 2; HIFs, hypoxia-inducible factors; HO-1, heme oxygenase-1. Created with BioRender.

Lipid peroxidation can be initiated by non-enzymatic and enzymatic processes [25]. The non-enzymatic process is triggered by Fenton reaction, where iron and hydrogen peroxide react toward free radical formation and propagation of lipid peroxidation [15]. Numerous enzymes were implicated in the regulation of ferroptosis and are outlined in Fig. 1. Some of the key enzymes are described below.

### Ferroptosis antagonists

Glutathione peroxidases (GPXs) protect cells from oxidative stress, and hence ferroptosis [14–16]. In fact, GPX4 is the main enzyme catalyzing the reduction of phospholipid peroxides to its corresponding phospholipid alcohol. This selenoprotein is regulated by a number of ncRNAs including miR-101-3p, miR-324-3p, lncPVT1, circCDK14, circKDM4C, and circDTL [26–32]. In addition, cofactor of GPX4 is glutathione, an antioxidant that uses cysteine for its synthesis [14–16]. In addition to the intracellular synthesis from methionine and glucose, and the interconversion of cysteine and homocysteine through intermediate cystathionine (transsulfuration pathway), cysteine can be imported from the environment [15]. The oxidized form (cystine) is imported by the system x_c_^-^ cystine/glutamate antiporter (see also Fig. 1). Subunits of this transmembrane protein complex are solute carrier family 7 member 11 (SLC7A11) and solute carrier family 3 member 2 (SLC3A2) [33]. Numerous ncRNAs regulate the expression of SLC7A11, including miR-375, miR-214-3p, miR-5096, LINC00618, P53RRA, OIP5-AS1, circPVT1, circEPSTI1 [34–41].

Cyst(e)ine-GSH-GPX4-system x_c_^-^ is considered as the main antagonist of ferroptosis. Ferroptotic death via inhibition of system x_c_^-^ and GPX4 can be induced by the compounds erastin and RSL3 [19, 20]. While RSL3 impairs GPX4 activity directly, erastin inhibits cystine import into the cell and thereby indirectly affects GPX4 activity. Erastin additionally targets voltage-dependent ion channels inducing mitochondrial dysfunction [19, 20]. RSL3 and erastin are frequently used in experimental approaches to induce ferroptosis.

Several other opposing mechanisms of ferroptosis have been described. Ferroptosis suppressor protein 1 (FSP1) reduces lipid peroxidation and ferroptosis by reducing the oxidized form of coenzyme Q10, ubiquinone, to yield ubiquinol, which then reduces lipid radicals and prevents propagation of lipid peroxidation [42]. Tetrahydrobiopterin (BH_4_) protects phospholipids from oxidative degradation by acting as an antioxidant and aids ubiquinone synthesis [43]. In addition, FSP1 may also indirectly reduce lipid peroxidation via regeneration of vitamin E, another strong antioxidant of lipids [44]. There is evidence that vitamin E (and possibly selenium) supplementation may promote cancer development [45]. FSP1 is indirectly upregulated by miR-4443, and several transcription factors including nuclear factor E2-related factor 2 (NRF2) [46, 47]. NRF2 is an important transcription activator that regulates various genes involved in metabolism, inflammation, mitochondrial respiratory, proliferative and transport processes [48]. In particular, NRF2 is crucial for cell survival during the oxidative stress. In addition to FSP1, it promotes the expression of several other negative regulators of ferroptosis, such as ferritin heavy chain 1 (FTH1), SLC7A11, and cystathionine β-synthase (CBS) (Fig. 1) [49–51]. Radical-trapping antioxidants ferrostatin 1 and liproxstatin 1 are useful in experimental studies to suppress ferroptosis [52].

### Ferroptosis agonists

Acyl-CoA synthetase long-chain family member 4 (ACSL4) and lysophosphatidylcholine acyltransferase 3 (LPCAT3) are essential drivers of ferroptosis (Fig. 1) [53]. ACSL4 is the important enzyme in lipogenesis. It catalyzes the reaction between long-chain PUFAs with coenzyme A to produce long-chain fatty acyl-CoA esters. These products are re-esterified into phospholipids by LPCAT3 enzyme, thus enhancing the incorporation of long-chain PUFAs into lipids and membranes [53]. MiR-23a-3p, miR-424-5p, and NEAT1 post-transcriptionally suppress, while circKDM4C upregulates expression of ACSL4 [32, 54–56]. Furthermore, reports suggest that certain arachidonate lipoxygenases (ALOXs) may contribute to ferroptosis via stereotactic insertion of oxygen in PUFAs [15], while lipoxygenase inhibitors act as radical-trapping antioxidants (Fig. 1) [15, 57, 58]. MiR-7-5p and miR-522 are known repressors of ALOX12 and ALOX15, respectively [59, 60]. Interestingly, *Pseudomonas aeruginosa* secretes lipoxygenases that can induce oxidation of membrane lipids of human red blood cells and induce ferroptosis in bronchial epithelial cells, a finding particularly important for cystic fibrosis patients who are susceptible to this bacterium [21, 22].

In addition, cytochrome P450 oxidoreductase may initiate lipid peroxidation by reducing ferric iron (Fe^3+^) to ferrous iron (Fe^2+^), a reaction that is crucial for the Fenton reaction and lipid peroxidation (Fig. 1) [61]. Although phospholipid peroxides may interact with both forms of iron, ferrous iron is likely to be more important since ferric iron has poor solubility and bioavailability in cells [14].

### Consequences of ferroptosis

Some of the main end products of this process are two omega-6 fatty acids, toxic 4-hydroxy-2-nonenal (4-HNE) and mutagenic malondialdehyde (MDA) (Fig. 1) [62]. Consequences of ferroptosis include formation of various secondary lipid peroxide breakdown products, modification and oxidation of proteins, and eventual breakdown of membrane integrity. Morphologically, ferroptotic cells have small mitochondria with increased mitochondrial membrane densities, reduced or vanishing mitochondrial cristae, and rupture of the outer mitochondrial membrane [52]. In addition, ferroptotic death may induce cell membrane rupture, release of intracellular content such as damage-associated molecular patterns (DAMPs), inducing sterile inflammation and can therefore be classified as a form of regulated necrosis [15]. Membrane repair is dependent on endosomal sorting complexes required for transport-III (ESCRT)-III (Fig. 1). This protein complex, consisting of 12 subunits, assembles into the spiral filament and mediates membrane remodeling [63].

### Ferroptosis, metabolic and cellular signaling pathways

The regulation of ferroptosis is strongly connected to various essential cellular processes, including metabolic pathways (iron, lipids, amino acids, and glucose metabolism), mitochondrial activity, maintenance of redox status, or response to radiation exposure. Furthermore, several key mediators of cell signaling pathways have been implicated in the regulation of ferroptosis, including multiple oncogenic and tumor-suppressive proteins (e.g., p53) [14, 16].

Iron metabolism plays a central role in ferroptosis. For example, transferrin and its receptor import iron into the cells and promote ferroptosis [64]. In contrast, mechanisms that export cellular iron have been shown to reduce ferroptosis [65, 66]. Autophagic degradation of major iron-storage protein ferritin promotes ferroptosis due to increased iron availability (Fig. 1). This process is named ferritinophagy [67]. Other types of autophagy, such as lipophagy, clockophagy and chaperone-mediated autophagy may also contribute to induction of ferroptosis via degradation of negative regulators of ferroptosis [68–70]. Indeed, there is growing evidence asserting the interaction between ferroptotic and autophagic machinery [71]. For example, lipid peroxidation products (i.e., 4-HNE) may induce autophagosome formation [72]. Heme oxygenase-1 (HO-1), a source of intracellular iron, is found to promote macroautophagy [73]. Erastin also promotes chaperone-mediated autophagy via upregulation of lysosome-associated membrane protein 2a (LAMP-2A), that may also degrade GPX4 [74]. In contrast, overexpression of GPX4 has been shown to inhibit ROS-mediated autophagy [75].

The activity of several metabolic pathways can affect the generation of ROS and are therefore strongly associated with the induction of ferroptosis. For example, glutamine can replenish tricarboxylic acid (TCA) cycle through the generation of α-ketoglutarate [64]. High glutamine uptake and metabolism can result in increased TCA cycle activity and increased rate of mitochondrial respiration, leading to ROS formation and loss of the mitochondrial membrane potential. High extracellular concentration of glutamate impedes system x_c_^-^ function due to inhibition of cystine uptake, and eventual intracellular glutathione synthesis, therefore leading to the ferroptosis induction [12]. Hypoxia promotes ferroptosis by increasing ROS production which can directly contribute to lipid peroxidation and activation of hypoxia-inducible factors (HIFs) [14]. HIFs have been shown to drive ferroptosis in clear cell renal carcinoma [76]. Even though glucose starvation increases ROS generation, it actually suppresses ferroptosis through the activation of energy sensor AMP activated kinase (AMPK). Energy-stress-mediated AMPK activation inhibits acetyl-CoA carboxylase which blocks the conversion of acetyl-CoA to malonyl-CoA and thus synthesis of PUFAs [77, 78]. In contrast, the core regulator of autophagosome formation Beclin-1 (BECN1) promotes ferroptosis by inhibiting system x_c_^-^ activity in energy-sufficient AMPK-mediated manner [79].

Tumor suppressor proteins may sensitize cells to ferroptosis. P53, an important regulator of apoptosis and autophagy, enhances ferroptosis and prevents tumor development via suppressing the transcription of system x_c_^-^ component SLC7A11 [80]. In addition, BRCA1-associated protein 1 (BAP1) also promotes ferroptosis by SLC7A11 downregulation [81]. Involvement of oncogenes in ferroptosis is best illustrated through the initial discovery of stronger lethality of erastin and RSL3 in RAS-mutated cancer cells, suggesting determining role of RAS-RAF-MEK pathway in ferroptosis [82].

**Fig. 2:**
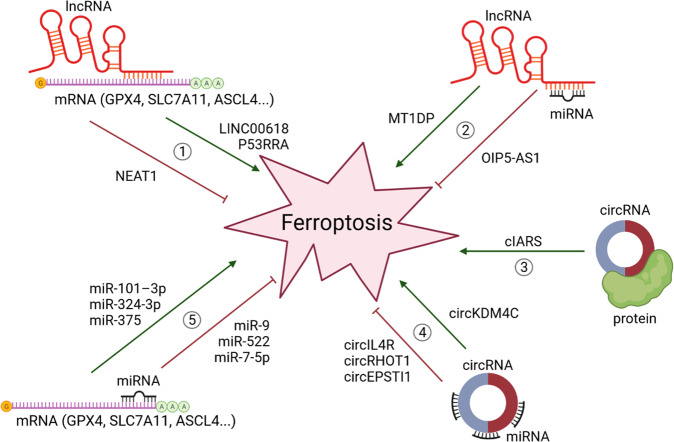
NcRNAs implicated in regulation of ferroptosis. NcRNAs regulate ferroptosis via modulation of its key players at mRNA and protein levels. Ferroptosis agonists (ASCL4, LPCAT3, ALOX, CYP450) and ferroptosis antagonists (GPX4, SLC7A11, SLC3A2, FSP1) can be sponged and inhibited by overexpressed lncRNAs (pathways 1 and 2), circRNAs (pathways 3 and 4), and miRNAs (pathway 5) thus regulating the ferroptosis activity. Created with BioRender.

Furthermore, ionizing radiation has been shown to upregulate ACSL4 expression in cancer leading to increased lipid peroxidation and ferroptosis [83]. Clearly, many more regulators of ferroptosis are yet to be discovered and characterized. As mentioned, and exemplified earlier in text, ncRNAs as versatile master regulators of cellular processes have also recently been linked to the regulation of ferroptosis (Fig. 2). The following sections will systematically summarize the current knowledge on the involvement ncRNAs in regulation of ferroptosis and their role in cancer.

### The role of miRNAs in the regulation of ferroptosis in cancer

MiRNAs are a class of small evolutionarily conserved ncRNAs with a length of approximately 22 nucleotides. Their main function is post-transcriptional regulation of gene expression through binding to complementary target mRNA sequences, leading to translational inhibition or mRNA degradation. The final result is halted protein synthesis [84, 85]. In addition, it has been reported that miRNAs may also induce gene expression by binding to target sequence and act as translational activator [86]. While they were completely unknown less than three decades ago, nowadays it has been estimated that miRNAs control the expression of over 60% of all protein-coding genes [87]. Given their substantial regulatory capacity, it seems obvious that deregulation of this tightly controlled miRNA network is frequently linked to cardiovascular, autoimmune, infectious, and neurodegenerative diseases [84]. In fact, they are increasingly recognized as major mediators of disease. The first link between miRNA and human cancer was reported in 2002 by Calin and colleagues [88]. Since then, thousands of miRNAs have been discovered, their deregulation in virtually every type of cancer has been confirmed, and their involvement in all hallmarks of cancer has been revealed [89]. Their regulatory roles in ferroptosis in cancer are not well understood yet, but there is strong evidence that miRNAs are also involved in this crucial process in cancer cells (Fig. 2). Some prominent examples are outlined below and a broader overview is provided in Table 1.

**Table 1:** Examples of miRNAs implicated in ferroptosis regulation in cancer.

miRNA	Role in ferroptosis	Mechanism of action	Reference
miR-375	Induces ferroptosis in gastric cancer	Downregulates SLC7A11	[34]
miR-4715-3p	Induces ferroptosis in gastric and esophageal carcinomas	Downregulates AURKA and GPX4 expression	[170]
miR-214-3p	Induces ferroptosis in HCC	Downregulates ATF4 and SLC7A11	[35]
miR-101-3p	Induces ferroptosis in lung cancer	Downregulates TBLR1, NF-κB, GPX4 and PTGS2 expression	[26]
miR-324-3p	Induces ferroptosis in LUAD	Downregulates GPX4	[27]
miR-324-3p	Induces ferroptosis in breast cancer	Downregulates GPX4	[28]
miR-5096	Induces ferroptosis in breast cancer	Downregulates SLC7A11	[36]
miR-1287-5p	Induces ferroptosis in osteosarcoma	Downregulates GPX4	[171]
miR-137	Inhibits ferroptosis in melanoma	Downregulates SLC1A5	[93]
miR-9	Inhibits ferroptosis in melanoma	Downregulates GOT1, inhibits glutaminolysis	[172]
miR-130b-3p	Inhibits ferroptosis in melanoma	Downregulates DKK1, upregulates NRF2 and HO-1 expression	[173]
miR-103a-3p	Inhibits ferroptosis in gastric cancer	Downregulates GLS2, prevents hydrolysis of glutamine to glutamate	[174]
miR-522	Inhibits ferroptosis in gastric cancer	Downregulates ALOX15	[60]
miR-23a-3p	Inhibits ferroptosis in HCC	Downregulates ACSL4	[54]
miR-4443	Inhibits ferroptosis in NSCLC	Downregulates m6A, upregulates FSP1	[46]
miR-424-5p	Inhibits ferroptosis in ovarian cancer	Downregulates ACSL4	[55]
miR-7-5p	Inhibits ferroptosis in ovarian, oral squamous cell and HCC	Downregulates mitoferrin, reduces mitochondrial iron levels	[99]
miR-7-5p	Inhibits ferroptosis in cervical and oral squamous carcinomas	Upregulates ferritin, downregulates *ALOX12* expression	[59]

### Ferroptosis-stimulating miRNAs

MiR-214-3p promotes ferroptosis in hepatocellular carcinoma (HCC) by downregulating the expression of activating transcription factor 4 (ATF4) [35]. ATF4 is induced by stress signals and prevents ferroptosis through the induction of SLC7A11 [90]. In addition, ATF4 regulates the expression of genes involved in differentiation, metastasis and angiogenesis [91]. In gliomas, ATF4 promotes tumor angiogenesis which can be diminished in vitro with ferroptosis inducers [92].

In lung cancer, miR-101-3p is found to be downregulated [26]. When available, this miRNA targets oncogenic transducin beta-like 1X-linked (TBLR1) protein. Low expression of miR-101-3p and high expression of TBLR1 result in enhanced activity of the transcription factor nuclear factor kappa B (NF-κB), which regulates ferroptosis through GPX4 and prostaglandin-endoperoxide synthase 2 (PTGS2). Interestingly, appealing results on tumor growth reduction were observed in in vivo experiments when miR-101-3p was delivered in the form of nanoparticles [26].

Moreover, miR-324-3p is significantly downregulated in lung adenocarcinoma (LUAD) cell lines when compared to healthy cells. When overexpressed, miR-324-3p induces ferroptosis by targeting GPX4, and enhances cisplatin sensitivity [27]. This miRNA is additionally upregulated by metformin in breast cancer cell lines, and in vivo experiments lead to GPX4 downregulation and ferroptosis induction [28].

### Ferroptosis-inhibitory miRNAs

It has been shown in melanoma cells that miR-137 inhibits lipid peroxidation and iron accumulation in vitro and in vivo by directly targeting solute carrier family 1 member 5 (SLC1A5) [93]. SLC1A5, a non-member of system x_c_^-^, is a neutral amino acid transporter of alanine, serine, cysteine, and glutamine [94]. As a consequence, reduced levels of this important glutamine transporter lead to decreased glutamine uptake, glutaminolysis, and MDA accumulation [93]. Under physiological conditions, glutamine uptake and its metabolism induce lipid ROS generation and ferroptotic cell death [64]. In addition, miR-137 is associated with TNM stage, metastasis and drug resistance in various cancers through different pathways [95–98].

In addition, miR-7-5p is highly expressed in radioresistant cell lines of ovarian, oral squamous cell and HCC. miR-7-5p downregulates mitoferrin, a protein responsible for transporting iron into mitochondria. Ferroptosis is diminished as a result of reduced iron levels [99]. This miRNA is also upregulated in radioresistant cervical cancer [59]. The observed radio-resistance in cervical and oral squamous carcinoma cell lines is, at least partly, due to miR-7-5p effect on ferroptosis. Knockdown of miR-7-5p is shown to increase ROS levels, mitochondrial membrane potential, intracellular Fe^2+^ content, as well as downregulation of the iron storage protein ferritin, and upregulation ALOX12 expression [59].

MiR-4443 is upregulated in non-small cell lung cancer (NSCLC) where it contributes to cisplatin resistance [46]. In addition, this miRNA may be transferred to the sensitive cells via exosomes and make them resistant. Mechanistically, miR-4443 inhibits ferroptosis via regulation of FSP1 expression in an N^6^-methyladenosine (m6A) manner by directly targeting its gene *METLL3* [46].

MiRNAs have extremely diverse regulatory roles. In addition to direct regulation of ferroptotic key players, as outlined in the above section, miRNAs may indirectly regulate this cell death process via interaction with other ncRNAs, as illustrated in the following sections.

### The role of lncRNAs in the regulation of ferroptosis in cancer

LncRNAs are a class of heterogeneous ncRNAs that are more than 200 nucleotides in length. They share many features with mRNA regarding transcriptional and post-transcriptional processing [100]. Despite being classified as ncRNA, the relevance of the protein-coding potential of lncRNA is growing [101]. Nevertheless, current evidence infers that lncRNAs mainly regulate cellular processes through the interaction with various other molecules, such as DNA, RNA, and proteins [102, 103]. Having a much broader interactome than miRNAs, lncRNAs can also control chromatin structure, methylation status, sequestration of miRNAs, assembly or disruption of protein complexes, and post-translational modifications [100, 103]. Another feature of lncRNAs is their tissue- and condition-specific (e.g., cancer-specific) expression pattern [100]. The first lncRNAs, H19 and Xist, were discovered in 1980s and 1990s, but they remained exceptions until the early 2000s when characterization of ncRNAs started to outpace protein-coding genes [104]. Dysregulated lncRNAs are involved in all hallmarks of cancers, including sustained angiogenesis and deregulated cellular metabolism [105, 106]. In addition, mounting evidence suggest their importance in ferroptosis regulation, as outlined below (Fig. 2).

### Ferroptosis-stimulating lncRNAs

Tumor suppressive lncRNA P53RRA, also known as LINC00472, is downregulated in various cancers including lung, liver, colon, renal and breast cancers [107–111]. In lung cancer, it interacts with Ras GTPase-activating protein-binding protein 1 (G3BP1) in the cytosol [38]. This cytosolic P53RRA–G3BP1 interaction displaces p53 from the G3BP1 complex. In turn, p53 is retained in the nucleus, leading to cell-cycle arrest, apoptosis, and ferroptosis. P53RRA promotes ferroptosis and apoptosis by affecting transcription of several metabolic genes, including the downregulation of SCL7A11. Additionally, P53RRA increases erastin-induced ferroptosis, lipid ROS and iron concentrations [38].

Furthermore, lncRNA GA binding protein transcription factor beta subunit 1 antisense RNA 1 (GABPB1-AS1) is upregulated by erastin in HCC cells. It inhibits the translation of GA binding protein transcription factor subunit beta 1 (GABPB1) protein, which acts as an activation subunit of transcription activator nuclear respiration factor 2, also called GA-binding protein (GABP). Downregulated GABPB1 protein leads to the downregulation of peroxiredoxin-5 peroxidase (PRDX5). The resulting suppression of the cellular antioxidant capacity causes accumulation of ROS and MDA, and reduction in cell viability [112].

Metallothionein 1D pseudogene (MT1DP) is a lncRNA that regulates erastin-induced ferroptosis through NRF2 [113]. Ectopic MT1DP expression in NSCLC upregulates ROS and MDA levels, increases intracellular ferrous iron concentration, and reduces glutathione levels in cancer cells exposed to erastin. These effects are achieved through downregulation of NRF2, indirectly via stabilization of miR-365a-3p that normally targets NRF2 mRNA. Interestingly, Gai and colleagues designed folate-modified liposome nanoparticles to enhance the bioavailability and the efficiency of the targeted delivery of both erastin and MT1DP. In vivo mice studies have shown promising results for erastin-induced ferroptosis through this particular pathway in NSCLC [113].

### Ferroptosis-inhibitory lncRNAs

LINC00336 is a nuclear lncRNA with oncogenic functions in lung cancer, including the regulation of ferroptosis [114]. It interacts with RNA-binding protein ELAV-like RNA-binding protein 1 (ELAVL1), which stabilizes the LINC00336 via binding adenylate and uridylate (AU)-rich elements (AREs), the signal regions that determine RNA stability. In addition, it is indirectly upregulated through the p53 signaling pathway since LSH increases ELAVL1 expression. When upregulated, LINC00336 acts as an endogenous sponge of miR-6852, thus preventing miRNA-induced downregulation of CBS. The result is inhibited ferroptosis in lung cancer cells, leading to enhanced cell proliferation, colony formation, and tumor formation. LINC00336 has been shown to decrease iron concentration, lipid ROS, and mitochondrial superoxide, and increases mitochondrial membrane potential [114].

Zhang et al. investigated the effects of chronic cadmium exposure - one of the causative factors of prostate cancer - on cellular growth and ferroptosis resistance in vitro and in vivo. After the cadmium exposure, the expression of ferroptosis-related proteins (particularly GPX4, FTH1 and SLC7A11) was increased, suggesting that cadmium exposure confers ferroptosis resistance. These effects were preceded by upregulation of lncRNA OIP5-AS1 expression. OIP5-AS1 acts as an endogenous sponge of miR-128-3p to regulate the expression of SLC7A11 [39].

Nuclear enriched transcript 1 (NEAT1) is a well-known oncogenic perinuclear lncRNA that has significant roles in non-cancerous diseases as well [115, 116]. It is associated with several hallmarks of cancers including proliferation, cell cycle, invasion, migration and apoptosis [117]. Wu et al. found that NEAT1 is capable of binding to ACSL4 mRNA, thus reducing the expression level of this pro-ferroptotic enzyme in NSCLC [56]. While NEAT1 contributes to apoptosis, its role in ferroptosis in NSCLC seems to be independent from it. Also, its contribution to ferroptosis is mediated exclusively via ACSL4 as erastin induction does not significantly affect other ferroptotic players, such as SLC7A11, GPX4, and TfR1 levels [56].

Table 2 provides additional lncRNAs involved in ferroptosis regulation in cancer.

**Table 2:** Examples of lncRNAs implicated in ferroptosis regulation in cancer.

lncRNA	Role in ferroptosis	Mechanism of action	Reference
P53RRA (LINC00472)	Induces ferroptosis in lung cancer	Downregulates SCL7A11	[38]
MT1DP	Induces ferroptosis in NSCLC	Stabilizes miR-365a-3p, downregulates NRF2	[113]
GABPB1-AS1	Induces ferroptosis in HCC	Inhibits GABPB1 translation, downregulates GABPB1 and PRDX5	[112]
LINC00618	Induces ferroptosis in leukemias	Downregulates SLC7A11 via attenuation of LSH expression	[37]
LINC00336	Inhibits ferroptosis in lung cancer	Stabilized by ELAVL1 and LSH. Sponges miRNA6852, upregulates CBS	[114]
NEAT1	Inhibits ferroptosis in NSCLC	Downregulates ACSL4 expression	[56]
H19	Inhibits ferroptosis in breast cancer	Inhibits production of lipid ROS and induces production of GSH	[175]
lncPVT1	Inhibits ferroptosis in HCC	Sponges miR-214-3p, upregulates GPX4	[29]
OIP5-AS1	Inhibits ferroptosis in prostate cancer	Sponges miR-128-3p, upregulates SLC7A11 expression	[39]
RP11-89	Inhibits ferroptosis in bladder cancer	Sponges miR-129-5p, upregulates PROM2 which induces iron export	[176]
MEG8	Inhibits ferroptosis in benign hemangioma	Sponged by miR-497-5p. Upregulates SLC7A11 and GPX4 expression	[177]

### Ferroptosis-related lncRNAs in the prediction of therapy responses and outcomes

In addition to the above-mentioned lncRNAs with confirmed regulatory mechanisms, RNAseq investigations associated many other lncRNAs with ferroptosis. For example, signatures consisting of eight to twelve differentially expressed lncRNAs were shown to be independent prognostic factors for overall survival (OS) in breast cancer [118, 119]. In the study from Zhang et al., patients with high-risk score had worse prognosis when treated with endocrine therapy, anthracycline, cyclophosphamide or paclitaxel, but not anti-HER2 therapy. In general, their tumors were immunologically cold due to inactivation of immune-related pathways and reduced tumor’s immune cells infiltration [119]. Similarly, Yao et al. correlated seven ferroptosis-related lncRNAs with clinical prediction of prognosis and immunotherapeutic responses in LUAD [120]. In the same fashion, Jian et al. developed ferroptosis-related lncRNAs signature for glioma consisting of 15 lncRNAs. They also showed that patients in high-risk group had lower tumor purity, higher infiltration of immunosuppressive cells, and higher expression of immune checkpoints. In addition, those patients had no survival benefits of radiotherapy, compared to the low-risk group [121].

In the first study investigating the roles of ferroptosis-associated lncRNAs in the prognosis of head and neck cancer (HNSCC), a total of 25 differently expressed lncRNAs were found to be independent prognosis factors for OS [122]. It was revealed that those novel ferroptosis-related lncRNAs in HNSCC may regulate immune and tumor-related pathways, particularly the expression of PD-1, CTLA4, LAG3, and BTLA [122]. Hence, combining immune checkpoint inhibitors with ferroptosis inducers may synergistically reduce cancer growth [123]. There is limited ongoing research that explores the relationship between immune checkpoints, radiotherapy and ferroptosis. Therefore, lncRNAs implicated in these processes should be investigated further. Nevertheless, all above-mentioned signature profiles need further validation using different cohorts and complete molecular characterization before having a potential of being used as biomarkers or targets for novel medications.

### The role of circRNAs in the regulation of ferroptosis in cancer

CircRNAs are single-stranded, covalently closed ncRNA molecules with distinct characteristics from other ncRNAs [124]. Their existence was first reported several decades ago. Initially, it was believed that they are merely splicing-associated noise that arises from irregular splicing and represents procedural errors. Therefore, their biological relevance was initially underappreciated [125].

Generally being classified as non-coding molecules, it has been found that some circRNAs have AUG sites and may be abundantly associated with polysomes [125, 126]. Similar to lncRNAs, circRNAs seem to be highly conserved and exhibit tissue-specific expression [127, 128]. Fairly contrary to the linear miRNAs and lncRNAs, circRNAs are exceptionally stable thanks to their circular nature, leaving them without free ends to be degraded by exonucleases. Due to their increased stability, circRNAs can be found in exosomes and extracellular fluids, such as saliva and plasma. Therefore, they have great biomarker potential [129].

While some circRNAs (e.g., intron-containing circRNAs) are found only in the nucleus where they might play a role in transcription regulation, most circRNAs are located in the cytoplasm [125]. There they usually function as miRNA sponges. Individual circRNAs can bind to multiple miRNAs that regulate different pathways [124, 125]. This feature is of particular importance for the potential therapeutic purposes. Importantly, miRNA-circRNA interactions might not always result in miRNA suppression, but also vice versa. Therefore, circRNAs may also function as miRNAs transportation or reservoir agents [124, 130]. However, growing caution stands for the alteration of miRNAs’ activity via sequestration by other ncRNAs (e.g., circRNAs and lncRNAs), the phenomenon called competing endogenous RNA (ceRNA) hypothesis [131]. Recent findings alert that physiological and even pathological changes in individual ceRNA expression are usually insufficient in significant miRNA activity suppression [132–134]. This limitation is based on the fact that individual ceRNA constitute only a small fraction of miRNAs’ large target pool. Moreover, mathematical models assert that optimal ceRNA inhibition occurs when miRNA and targets are at near equimolar concentrations [135–137]. Many previously published studies have used supraphysiologic concentrations of transfected oligonucleotides or expression vectors that frequently exceed total cellular concentrations of their natural counterparts. This clearly suggests an overestimation of ceRNA activity and demand for better molecular models. Lastly, ceRNA as the appealing and straightforward approach in ncRNA studying may potentially hinder researchers’ consideration of other confirmed ncRNAs’ mechanisms of action. Nevertheless, circRNAs are important regulators of numerous normal and pathological cellular processes and diseases, including cancer [138]. So far, circRNAs have been associated with several hallmarks of cancers, including sustained proliferative signaling, evasion of growth suppressors, angiogenesis, invasion and metastasis, and evading cell death and senescence [124, 138]. Growing evidence associate them with ferroptosis (Fig. 2).

### Ferroptosis-stimulating circRNAs

Three circRNAs capable of ferroptosis induction are cIARS, circKDM4C and circ_0000190. cIARS is derived from the *IARS* gene, and it is found to be highly expressed in HCC after sorafenib treatment. Liu et al. found that it promotes ferroptosis after sorafenib treatment through, at least partially, activation of autophagy and ferritinophagy. This circRNA physically interacts with RNA binding protein AlkB Homolog 5 (ALKBH5) [139]. ALKBH5 is known for the improvement of Bcl-2 mRNA stability by catalyzing m6A demethylation, thus enhancing Bcl-2/BECN1 interactions. It is also known as autophagy inhibitor in cancer [140]. Sorafenib administration increases cIARS*–*ALKBH5 interaction, which is probably due to sorafenib-induced expression of cIARS as this kinase inhibitor has no influence on the ALKBH5 protein levels. Consequently, cIARS represses negative role of ALKBH5 in autophagy leading to enhanced autophagy, ferritinophagy and ferroptosis [139]. Up to date, cIARS is the only circRNA that regulates ferroptosis via interaction with a formed protein.

Further, circKDM4C is downregulated in AML. Normally, it sponges miRNA let-7b-5p which targets p53. In addition to indirect upregulation of p53, circKDM4C, when not retrieved from the circRNA pool, is capable of ferroptosis induction via increasing cellular iron content, upregulation of ACSL4 and PTGS2, and downregulation of GPX4 and FTH1 [32].

### Ferroptosis-inhibitory circRNAs

Xian et al. found that circular ATP binding cassette subfamily B member 10 (circABCB10) is upregulated in colorectal cancer (CRC) where it acts as a sponge to miR-326. Consequently, its target C-C motif chemokine ligand 5 (CCL5) is overexpressed and contributes to carcinogenic effects, including inhibition of apoptosis and ferroptosis [141]. CCL5 has already been associated with CRC development and progression [142]. However, its exact mechanism in ferroptosis is still not reported.

Furthermore, circEPSTI1 is upregulated in cervical cancer contributing to enhanced cellular proliferation. Mechanistically, circEPSTI1 is capable of sponging three miRNAs, namely miR-375, miR-409-3p and miR-515-5p. All three of those miRNAs normally target SLC7A11, that acts as a ferroptosis inhibitor [41]. Other circRNAs that upregulate SLC7A11 are circ0097009 (sponges miR-1261 in HCC) [143], circ_0067934 (sponges miR-545-3p in thyroid cancer) [144], circCDK14 (sponges miR-3938 in glioma) [30], and circPVT1 (sponges miR-30a-5p in esophageal cancer) [40].

Moreover, GPX4 is, as mentioned above, considered to be pivotal regulator of ferroptosis, analogous to bcl-2 in apoptosis [145]. CircKIF4A, upregulated in papillary thyroid cancer, acts as a sponge of miR-1231, leading to GPX4 overexpression [146].

Table 3 provides an overview of additional circRNAs involved in ferroptosis regulation in cancer.

**Table 3:** Examples of circRNAs implicated in ferroptosis regulation in cancer.

circRNA	Role in ferroptosis	Mechanism of action	Reference
cIARS	Induces ferroptosis and ferritinophagy in sorafenib-treated HCC	Interacts with ALKBH5, improves Bcl-2 mRNA stability, enhances Bcl-2/BECN1 interaction	[139]
circ_0000190	Induces ferroptosis in gastric cancer	Sponges miR-382-5p, upregulates ZNRF3, inhibits Wnt/β-catenin signaling	[178]
circKDM4C	Induces ferroptosis in AML	Sponges miRNA let-7b-5p. When present, upregulates ACSL4, PTGS2 and p53, and downregulates GPX4 and FTH1	[32]
circ-TTBK2	Inhibits ferroptosis in glioma	Sponges miR-761, upregulates ITGB8 expression	[179]
circCDK14	Inhibits ferroptosis in glioma	Sponges miR-3938, upregulates PDGFRA, GPX4 and SLC7A11 expression	[30]
circKIF4A	Inhibits ferroptosis in papillary thyroid cancer	Sponges miR-1231, upregulates GPX4 expression	[146]
circ_0067934	Inhibits ferroptosis in papillary and follicular thyroid cancers	Sponges miR-545-3p, upregulates SLC7A11 expression	[144]
circDTL	Inhibits ferroptosis in NSCLC	Sponges miR-1287-5p, upregulates GPX4	[31]
circRHOT1	Inhibits ferroptosis in breast cancer	Sponges miR-106a-5p, upregulates STAT3	[180]
circGFRA1	Inhibits ferroptosis in breast cancer	Sponges miR‐1228, upregulates AIFM2 and GPX4 expression	[181]
circPVT1	Inhibits ferroptosis in esophageal cancer	Sponges miR-30a-5p, upregulates FZD3, GPX4 and SLC7A11 expression	[40]
circIL4R	Inhibits ferroptosis in HCC	Sponges miR-541-3p, upregulates GPX4 expression	[182]
circ0097009	Inhibits ferroptosis in HCC	Sponges miR-1261, upregulates SLC7A11	[143]
circABCB10	Inhibits ferroptosis in CRC	Sponges miR-326, upregulates CCL5	[141]
circ_0007142	Inhibits ferroptosis in CRC	Sponges miR-874-3p, upregulates GDPD5	[183]
circEPSTI1	Inhibits ferroptosis in cervical cancer	Sponges miR-375, miR-409-3p and miR-515-5p, upregulates SLC7A11 expression	[41]

### Ferroptosis and ncRNAs interplay: therapeutic potential in cancer

Remarkably, this form of cell death has been associated with numerous pathologic processes including neurodegeneration, liver and lung fibrosis, ischemia-reperfusion injuries in brain, heart, kidneys and organ transplantation [14, 16]. Nevertheless, there is major evidence of its particular relevance in cancer. It has been shown that mesenchymal and dedifferentiated cancer cells, which are resistant to cancer therapeutics and apoptosis, are highly susceptible to ferroptosis inducers [147, 148]. Hence, inducing ferroptotic cell death (e.g., by pharmacologic manipulation) may help to overcome resistance of malignant cells to chemotherapy and therefore has great potential for cancer treatment. Several strategies to specifically induce ferroptosis are already being tested. One option is to target key enzymes involved in ferroptosis in cancer cells. For example, pharmacologic and genetic inhibition of system x_c_^-^ by blocking SLC3A2 and SLC7A11 have shown promising results in mouse models with low toxicity [149–152]. Similarly, targeting FSP1 is a promising approach due to its irrelevance in normal mice development indicating a potential broad therapeutic window [42, 153].

While GPX4 is expressed in most cancer cell lines, it is essential for various organs, including kidneys and neurons [145, 154, 155]. Therefore, GPX4 inhibitors (e.g., RSL3) should be delivered specifically to the cancer cells to prevent side effects. Indirect ferroptosis inducers such as erastin may have low solubility and labile metabolism in the complex human body [156]. Incorporation of ferroptotic inducer compounds into protective delivery systems, such as nanoparticles, may overcome this problem. In addition, nanoparticles delivering iron, peroxides, and ncRNAs targeting key inhibitors of ferroptosis into cancer cells are already actively being tested in vitro and in vivo studies. NcRNAs are notably emerging as they ultimately carry several advantages. They are naturally occurring molecules in cells meaning their therapeutic counterparts may utilize existing cellular metabolic pathways. Additionally, ncRNAs frequently target multiple genes within one and/or more pathways causing a broader yet specific anti-cancer response, such as the case with miR-15 and miR-16 cluster that regulates various anti-apoptotic and cell cycle players, including bcl-2, mcl1 and c-JUN [157]. Lastly, ncRNA therapeutics can be fairly easy chemically synthesized shaping them as cost-effective medications of the future.

**Fig. 3:**
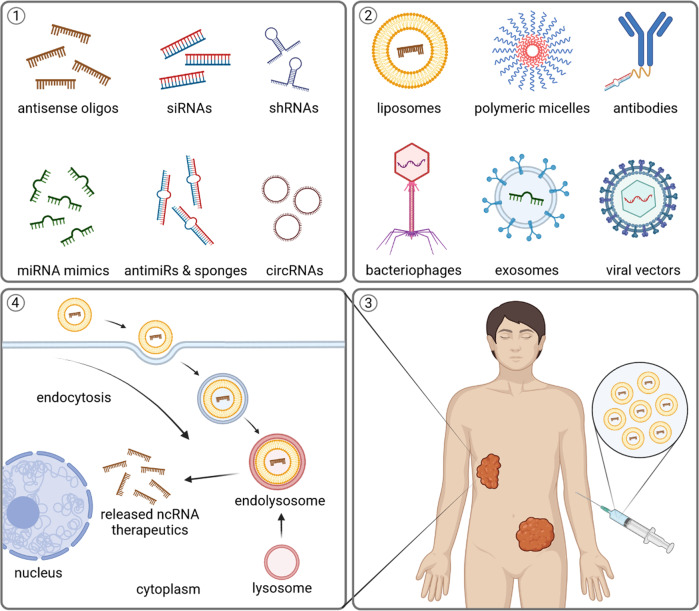
NcRNA-based therapeutics and delivery systems. Several ncRNA-based therapeutics exist, including antisense oligonucleotides, siRNAs, shRNAs, miRNA mimics, anti-miRNAs, miRNA sponges, and therapeutic circRNAs (box 1). Their major limitations and side effects can expectedly be overcome by usage of unique delivery systems that include lipid and polymer nanoparticles, antibodies, bacteriophages, exosomes and viral vectors (box 2). Newer generation of ncRNA therapeutics with convenient clinical administration (box 3) are awaited candidates due to their numerous advantages, including utilization of existing cellular processing mechanisms, capability of multiple signaling pathway targeting and promising cost-effective production (box 4). siRNA, small interfering RNA; shRNA, short hairpin RNA; antimiRs, anti-microRNAs; circRNAs, circular RNAs. Created with BioRender.

**Fig. 4:**
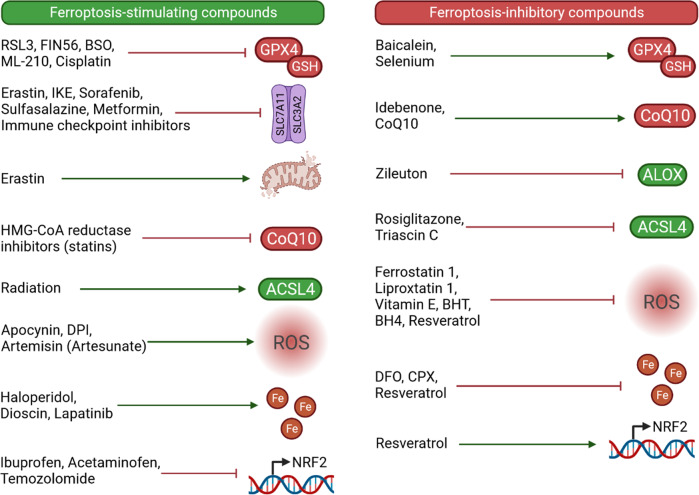
Compounds implicated in modulating ferroptosis. Some of these compounds are clinically available therapeutics that may additionally induce ferroptosis and/or sensitize cells to ferroptosis (cisplatin, metformin, sulfasalazine, sorafenib, immune checkpoint inhibitors HMG-CoA reductase inhibitors, ionizing radiation, artesunate, haloperidol, lapatinib, ibuprofen, acetaminophen, temozolomide), while others may prevent ferroptosis (selenium, idebenone, CoQ10, zileuton, rosiglitazone, vitamin E, DFO, CPX, resveratrol). In addition, several compounds currently limited to experimental studies carry potential for optimization toward clinical utilization in ferroptosis induction (erastin, RSL3, FIN56, BSO, ML-210, IKE, apocynin, DPI, dioscin) and ferroptosis blockade (baicalein, triascin C, ferrostatin 1, liproxstatin 1, BHT, BH4). RSL3, RAS synthetic lethal 3; BSO, buthionine sulfoximine; IKE, imidazole ketone erastin; DPI, diphenyleneiodonium chloride; BHT, butylated hydroxytoluene; BH4, tetrahydrobiopterin; DFO, deferoxamine; CPX, ciclopirox olamine. Created with BioRender.

Indeed, several ncRNA-based therapies are currently developed, including antisense oligonucleotides, small interfering RNAs, short hairpin RNAs, miRNA mimics, miRNA sponges, anti-microRNAs (antimiRs), and therapeutic circular RNAs (Fig. 3) [158, 159]. Some of them are targeting up-regulated oncogenic molecules, while others replenish downregulated tumor suppressors. Although majority are still being tested in clinical studies, eleven ncRNA-based therapeutics have already been approved for several other disease entities [159]. Major current limitations of ncRNA-based therapeutics are specificity, delivery, and tolerability. Issues with specificity and off-target effects occur due to uptake by the untargeted cells. The unstable nature of ncRNAs leads to their inefficient intracellular delivery, and adverse immune responses occur if ncRNAs are recognized as foreign viral nucleic acids [158–160]. Nevertheless, newer generations of RNA therapeutics are being designed to overcome those limitations and increase their chances of eventual clinical utilization. For example, it has been shown that circularization of small RNAs mediates more efficient and longer inhibiting effects on their targets, and overcome major current limitations of ncRNA-based therapeutics [161–163]. Particular advances have been made with chemical modifications and optimization of delivery methods that include lipid nanoparticles, polymers, antibodies, bacteriophages, and exosomes (Fig. 3) [159]. Furthermore, immune checkpoint inhibitors, radiotherapy and several medications including sorafenib, sulfasalazine, metformin, artesunate, temozolomide, cisplatin, may all induce ferroptosis and/or sensitize cells to ferroptosis (Fig. 4) [83, 164–169]. These findings propose the potential drug repositioning and synergistic combinatorial therapeutic regimens in the future. Figure 4 highlights the compounds, including available therapeutics, with their suggested roles in ferroptosis.

## Conclusion

Recently characterized as unique form of regulated cell death, ferroptosis has already been associated with numerous diseases – above all with cancer. However, our knowledge about ferroptosis is still fairly limited and many open questions remain. We still do not know the complete relationship between ferroptosis and other forms of regulated cell death that share some common upstream mechanisms, such as p53. In addition, redox-independent roles of iron as well as the roles of other metals (e.g., copper) are not completely ruled out in ferroptosis induction. In addition, the exact molecular events responsible for the execution of cell death via ferroptosis are not fully understood. This is particularly pronounced in our ignorance of molecular events that occur downstream of lipid peroxidation including crucial moment(s) when activated ferroptosis cannot longer be suppressed. Finally, specific markers of ferroptosis suitable for application in live cells and intact tissues are still lacking. Furthermore, ncRNAs are a heterogenous group of non-coding transcripts with exceptional regulatory and biomarker capacities. Only a fraction of annotated ncRNAs have been investigated in the context of ferroptosis and cancer. Nevertheless, current evidence suggests that ferroptosis is frequently inhibited in cancer through the deregulation of usually tightly controlled ncRNA networks, thereby aiding cancer cell survival and progression. Hence, artificial induction of ferroptosis carries a great therapeutic potential. Albeit in their infancies, emerging innovative discoveries in both fields are paving the exciting path toward the successful utilization of novel ferroptosis-modulating ncRNA-therapeutics in cancer.

## Data Availability

Correspondence and requests for materials should be addressed to Martin Pichler.
